# ICAM-1 levels in patients with covid-19 with diabetic foot ulcers: A prospective study in southeast asia

**DOI:** 10.1016/j.amsu.2021.02.017

**Published:** 2021-02-10

**Authors:** Mendy Hatibie Oley, Maximillian Christian Oley, Billy Johnson Kepel, Djony E. Tjandra, Fima Lanra Fredrik G. Langi, Herwen, Deanette Michelle R. Aling, Angelica Maurene Joicetine Wagiu, Muhammad Faruk

**Affiliations:** a Plastic Reconstructive and Aesthetic Surgery Division, Department of Surgery, Faculty of Medicine, Sam Ratulangi University, Manado, Indonesia; b Plastic Reconstructive and Aesthetic Surgery Division, Department of Surgery, R. D. Kandou Hospital, Manado, Indonesia; c Hyperbaric Centre Siloam Hospital, Manado, Indonesia; d Neurosurgery Division, Department of Surgery, Faculty of Medicine, Sam Ratulangi University, Manado, Indonesia; e Neurosurgery Division, Department of Surgery, R. D. Kandou Hospital, Manado, Indonesia; f Department of Chemistry, Faculty of Medicine, Sam Ratulangi University, Manado, Indonesia; g Vascular Surgery Division, Department of Surgery, Faculty of Medicine, Sam Ratulangi University, Manado, Indonesia; h Vascular Surgery Division, Department of Surgery, R. D. Kandou Hospital, Manado, Indonesia; i Department Epidemiology and Biostatistics, Public Health Faculty, Sam Ratulangi University, Manado, Indonesia; j Department of Surgery, Faculty of Medicine, Sam Ratulangi University, Manado, Indonesia; k Department of Surgery, R. D. Kandou Hospital, Manado, Indonesia; l Department of Surgery, Faculty of Medicine, Hasanuddin University, Makassar, Indonesia

**Keywords:** Intracellular adhesion molecule-1, Diabetic foot ulcers, COVID-19, Debridement

## Abstract

**Background:**

Viral infection can compound the severity of pre-existing inflammation caused by underlying diseases. For those with a chronic, immune-related condition such as diabetic foot ulcers (DFUs), the coronavirus disease (COVID-19) serves to exacerbate the inflammatory burden. Serum levels of intracellular adhesion molecule-1 (ICAM-1), a primary mediator of cell adhesion express in the inflammatory process, are often used to indicate the gravity of all inflammatory conditions. Therefore, the purpose of this study was to investigate serum ICAM-1 levels before and after debridement in patients with DFUs who were also diagnosed as COVID-19 positive compared with those who were COVID-19 negative.

**Methods:**

20 patients with DFUs were screened for COVID-19 and then divided into COVID-19 positive and negative groups according to the results. Before debridement, chest x-rays and blood analysis, including ICAM-1 serum levels, were performed in both groups. Only ICAM-1 serum levels were measured after debridement.

**Results:**

Of the 20 patients included in this study, 55% were male (n = 11) and 45% were female (n = 9). The mean age was 52.9 ± 1.9 years. ICAM-1 levels in patients with DFU in the COVID-19-positive group were significantly higher than those in the COVID-19-negative group (median 317.2 vs 149.2, respectively; p < 0.001). Serum levels of ICAM-1 reduced significantly in patients with DFU in the COVID-19-positive group were significantly higher than those in the COVID-19-negative group after debridement (median 312.5 vs 130.3; p < 0.001).

**Conclusion:**

ICAM-1 serum levels represent an additional, initial screening marker for COVID-19.

## HIGHLIGHTS

• Coronavirus Disease 2019 (COVID-19) is an infectious disease caused by SARS-CoV-2.
• In patients with diabetes, hyperglycemia causes both the innate and adaptive immune responses.
• ICAM-1 facilitates leukocyte-endothelial binding and the migration of leukocytes across the endothelial barrier.
• ICAM-1 levels in patients with DFU in the COVID-19-positive group were significantly higher than the COVID-19-negative group.
• ICAM-1 serum levels represent an additional, initial screening marker for COVID-19.

## 1 Introduction

Pre-existing inflammation caused by underlying diseases worsens in the presence of a viral infection. Coronavirus disease 2019 (COVID-19) is caused by infection with the severe acute respiratory syndrome coronavirus 2 (SARS-CoV-2). The common symptoms are fever, cough, dyspnea that can progress to pneumonia, acute respiratory distress syndrome, multiple organ failure, and death [[1], [2], [3]]. The first case of a ‘pneumonia of unknown etiology’ was reported on December 31, 2019, in Wuhan, Hubei Province, China by the World Health Organization (WHO) country office in China. The second case of coronavirus pneumonia was detected on January 7, 2020, and by March 11, 2020, the WHO declared COVID-19 a global pandemic. As of September 30, 2020, global COVID-19 cases reached 33,249,563 with a mortality of 1,000,040 people. Indonesia reported 278,722 COVID-19 cases, with 10,473 deaths [4,5].

Diabetes is one of the known comorbidity factors for COVID-19 [6,7]. The prevalence of diabetes mellitus increased from 108 million in 1980 to 422 million in 2014 according to the WHO. In Southeast Asia, the prevalence was 71.4 million in 2011 and this is predicted to increase to 120 million people in 2030 [8,9]. Diabetes mellitus is a syndrome that includes metabolic and vascular aspects in which fasting, postprandial, or random blood glucose levels increase. It can be classified into two types, type 1 diabetes mellitus (insulin-dependent) and type 2 diabetes mellitus (non-insulin-dependent). Complications from type 2 diabetes mellitus are grouped into two categories, microvascular (e.g., diabetic retinopathy, diabetic nephropathy, and diabetic neuropathy) and macrovascular (e.g., coronary vascular disease, peripheral vascular disease, brain vascular disease) [[10], [11], [12]]. Diabetic foot ulcers (DFUs) occur in more than 25% of patients with diabetes. It causes infection in 50% and amputation in 20% of patients. The number of lower extremity amputations is 10-20-fold higher in patients with diabetes compared with non-diabetics. Approximately 70–80% of all non-traumatic amputations are caused by diabetes. Patients with DFUs, therefore, undergo debridement for infection control to prevent amputation [13,14].

Patients with DFUs are exposed to prolonged, low-grade inflammation and infection. Triggered by inflammation, endothelial adhesion molecules such as ICAM-1 are produced in excessive amounts as a component of the immune response. Adding weight to the load, patients with DFUs who are also diagnosed with COVID-19 experience an acute inflammatory response on top of their pre-existing chronic inflammation. We hypothesize that ICAM-1 levels are elevated in these patients even after debridement. Therefore, this study aims to investigate the levels of ICAM-1 before and after debridement in patients with DFUs who are also diagnosed as COVID-19 positive compared with those who are COVID-19 negative.

## 2 Methods

This cohort prospective study was conducted at the Surgery Department of Prof. Dr. R.D. Kandou Hospital Manado from March to June 2020. This study was approved by the ethics commission for research at Prof. Dr. R.D. Kandou Hospital Manado (license no. 039/EC-KEPK/V/2020) and has been registered with the Research Registry (no. 6341). This study is reported in line with the Strengthening the Reporting of Cohort Studies in Surgery (STROCSS) guidelines [15].

### 2.1 Population and sampling

The population included in this study were patients with type 2 diabetes mellitus and DFUs with asymptomatic COVID-19 either positive or negative COVID-19 by RT-PCR of nasopharyngeal swab results. The inclusion criteria were: type 2 diabetes mellitus (random blood glucose > 200 mg/dL and HbA1c > 6.0%) with DFUs currently or previously receiving holistic therapy for diabetes mellitus, an ankle-brachial index over 0.9, HbA1c levels below 8%, and hemoglobin levels above 7.5 g/dL%. Individuals with a type 1 diabetes mellitus, traumatic ulcer, malignancy, coagulopathy, or any peripheral vascular disease were excluded from this study. The 20 remaining patients were screened for COVID-19 according to the hospital's COVID-19 protocol and Indonesian health protocols. The COVID-19 IgG/IgM Rapid Diagnostic Test identified 10 people as reactive and 10 as non-reactive for SARS-CoV2 antibodies.

The 10 reactive patients were tested for COVID-19 by RT-PCR of nasopharyngeal swab samples and 8 of these patients tested positive. The patients were then divided into two groups, 8 in the COVID-19 group and 12 in the non-COVID-19 group. Patients provided their written informed consent after receiving information about the advantages and risks of participating in the study.

### 2.2 Procedure

Before debridement, patients with DFUs were screened for COVID-19 using the IgG/IgM Rapid Diagnostic Test followed by an RT-PCR swab test if the former was reactive. Other tests included a chest x-ray (pneumonia), blood test [complete blood count, neutrophil to lymphocyte ratio (NLR)], liver function test (SGOT, SGPT), renal function test (urea, serum creatinine), and serum electrolyte (natrium, kalium, chloride), albumin, and ICAM-1 levels.

All patients underwent baseline digital anteroposterior chest radiography at full inspiration using a chest radiographic instrument (RADspeed Pro style edition MC, Shimadzu Corporation, Kyoto, Japan).

Blood was drawn from the peripheral vein before and after debridement. The COVID-19 IgG/IgM Rapid Diagnostic Test was applied immediately with the SARS-CoV-2 Antibody Test (Lateral Flow Method) from Guangzhou Wondfo Biotech Co Ltd (Guangzhou, China); while the complete blood counts and NLR were determined at our institution's clinical laboratory using a Sysmex XP-300™ (Sysmex; Kobe, Japan) according to the manufacturer's instructions. Serum electrolytes, liver function, renal function, and albumin were measured at our institution's laboratory using the Cobas® 8000 (Roche Diagnostics, Indianapolis, IN, USA) according to the manufacturer's instructions.

Nasopharyngeal swabs were sent in a protected container directly to the laboratory for COVID-19 RT-PCR tests with Abbott RealTime SARS-CoV-2 Assay from Abbott Molecular Inc, (Salt Lake City, IL, USA) according to the manufacturer's instructions [[16], [17], [18]]. ICAM-1 serum levels were measured 1-week before and after debridement according to the protocol in the Human sICAM-1 ELISA Kit (Merck Millipore, cat. no. ECM335, Burlington, Massachusetts, USA) [9,19,20]. All patients were then treated with a protocol for DFUs that included debridement. Blood samples were stored at 2-5 °C before being centrifuged (1000 rpm) for 60 min until coagulation occurred. The resulting samples were stored at −80 °C until further analysis.

The typical range for leucocytes is 4.0–10.0 × 10^3^/μL, erythrocytes 4.70 to 6.10 × 10^3^/μL, hemoglobin 13.0–16.5 g/dL, natrium 135 to 153 mEq/L, kalium 3.50 to 5.30 mEq/L, chloride 98.0 to 109.0 mEq/L, SGOT <33 U/L, SGPT <43 U/L, urea 10–40 mg/dL, serum creatinine 0.5–1.5 mg/dL, and albumin 3.50–5.70 g/dL.

### 2.3 Statistical analysis

Results from this study were presented as descriptive and analytical data. The descriptive tabulation of the patients' characteristics was carried out according to the type of variable. Normally distributed data are presented as a mean and standard deviation. If the distribution was not normal, median and interquartile range (IQR) were used for numerical variables and proportions for categorical variables. The *t*-test (or Mann-Whitney *U* test) and chi-square test (or Fisher's exact test) were used to correlate patient characteristics with their RT-PCR test results. Univariable distribution was presented using charts (histogram, boxplot, and Q-Q plot) for numerical data and a bar chart for categorical data. The Shapiro-Wilk test was used to determine the normality of the distribution of numerical variables. Serum ICAM-1 levels were analyzed based on their relationship with the RT-PCR swab test results for predicting accuracy in COVID-19 infection. The non-parametric Mann-Whitney *U* test was used to differentiate serum ICAM-1 levels from the COVID-19 and non-COVID-19 groups. Repeated measure analysis of variance (ANOVA) was used to determine the effect of time and the interaction between groups and time. All data were processed and analyzed using R version 4.0.1 software. Descriptive tabulation, graphing, and regression modeling were performed using the software's packages. Data were prepared using Microsoft Excel 2017.

## 3 Results

The 20 patients with DFUs who participated in this study underwent the RT-PCR swab test and were categorized into two groups, those with COVID-19 and those without. The RT-PCR swab test identified 12 positive and 8 negative samples. The mean age was 53 years and there was no difference in terms of age between the two groups. The sex ratio was almost equal, with slightly more men diagnosed with COVID-19 than women and no significant differences among those in the non-COVID-19 group. Anemia was seen in both groups, with a mean hemoglobin level of 10.01 g/dL. Leukocyte counts for both groups indicated leucocytosis, with the median value from the COVID-19 group being slightly lower than from the non-COVID-19 group; however, this difference was not statistically significant (12.9 vs 16.3; p = 0.280). The median platelet counts for both groups were within normal limits. The NLR had a median over 3.13 with no significant difference observed between the two groups, although the value from the non-COVID-19 group was higher (Table 1). The same was found for serum electrolyte levels, such as sodium, potassium, and chloride. There was no significant difference in the random blood sugar levels between the COVID-19 and non-COVID-19 groups (p = 0.323). Liver function parameters (SGPT and SGOT) were within the normal limits for both groups. Renal function tests (urea and serum creatinine) in the COVID-19 group appeared higher than the non-COVID-19 group (55.5 mg/dL and 1.9 mg/dL vs 33.5 mg/dL and 1.1 mg/dL). In contrast, serum albumin levels in the non-COVID-19 group indicated hypoalbuminemia (mean albumin serum 2.5 ± 0.7 g/dL) and were lower than the COVID-19 group (mean albumin serum 3.2 ± 0.5 g/dL; p = 0.027).

**Table 1:** Patient characteristics.

Characteristics	Total (N = 20)		Negative RT-PCR swab test (n = 12)		Positive RT-PCR swab test (n = 8)		p^a^
	Mean ± SD	Median (Q_1_; Q_3_)	Mean ± SD	Median (Q_1_; Q_3_)	Mean ± SD	Median (Q_1_; Q_3_)	
Age	52.9 ± 1.9	*	52.8 ± 115	*	52.9 ± 12.9	*	0.986
Sex, n (%)							
Male	11 (55)	*	5 (41.6)	*	6 (75)	*	0.370
Female	9 (45)	*	7 (58.3)	*	2 (25)	*	
Hemoglobin (g/dL)	10.1 ± 2.7	*	10.1 ± 1.9	*	10.1 ± 3.4	*	0.911
Leukocyte (10^3^ = mL)	*	15.9 (8.9; 26.7)	*	16.3 (13.3; 30.5)	*	12.9 (7.8; 20.8)	0.280
Erythrocyte (10^6^ = mL)	3.7 ± 1.0	*	3.7 ± 0.7	*	3.6 ± 1.3	*	0.870
Hematocrit (%)	29.6 ± 7,7	*	29.7 ± 5.7	*	29.4 ± 9.6	*	0.940
Thrombocyte (10^3^ = mL)	*	372.0 (232.8; 471.2)	*	323.0 (216.2; 483.5)	*	372.5 (251.2; 437.5)	0.912
Eosinophil (%)	*	1.0 (0.0; 3.0)	*	1.0 (0.0; 1.8)	*	2.0 (0.0; 3.8)	0.432
Neutrophil (%)	71.3 ± 11.7	*	72.7 ± 9.5	*	69.9 ± 13.9	*	0.605
NLR	*	3.5 (2.5; 6.2)	*	4.2 (2.8; 5.6)	*	3.4 (2.4; 10.4)	0.623
Lymphocyte (%)	18.1 ± 8.1	*	17.7 ± 6.9	*	18.4 ± 9.5	*	0.853
Monocyte (%)	6.5 ± 2.6	*	6.4 ± 2.1	*	6.7 ± 3.2	*	0.806
MCH (pg)	27.7 ± 1.6	*	27.1 ± 1.2	*	28.3 ± 1.7	*	0.069
MCHC (%)	34.1 ± 1.7	*	33.7 ± 1.9	*	34.5 ± 1.4	*	0.310
MCV (fL)	81.5 ± 5.2	*	80.6 ± 3.3	*	82.3 ± 6.7	*	0.493
Natrium (mg/dL)	132.5 ± 9.7	*	128.7 ± 11.2	*	135.2 ± 8.1	*	0.184
Kalium (mg/dL)	4.8 ± 1.7	*	4.6 ± 0.9	*	4.9 ± 2.1	*	0.757
Chloride (mg/dL)	95.2 ± 9.9	*	92.3 ± 11.8	*	97.3 ± 8.5	*	0.320
Random blood glucose (mg/dL)	196.8 ± 124.2	*	225.0 ± 147.4	*	168.6 ± 95.2	*	0.323
SGOT (mg/dL)	*	28.0 (18.0; 44.0)	*	21.0 (14.8; 41.2)	*	35.0 (28.0; 41.0)	0.270
SGPT (mg/dL)	*	18.0 (13.0; 21.0)	*	13.0 (11.5; 18.0)	*	19.0 (18.0; 22.0)	0.101
Urea (mg/dL)	*	40.5 (25.0; 140.8)	*	1.9 (0.7; 2.3)	*	33.5 (19.8; 166.2)	0.880
Creatinine (mg/dL)	*	1.5 (0.7; 3.5)	*		*	1.1 (0.7; 75)	0.472
Albumin (g/dL)	2.8 ± 0.7	*	2.5 ± 0.7	*	3.2 ± 0.5	*	0.027

Abbreviations: SD = standard deviation, Q1 = Quartile I, Q3 = Quartile III, SGOT = serum glutamic oxaloacetic transaminase, SGPT = serum glutamate-pyruvate transaminase, MCH = mean corpuscular hemoglobin, MCHC = mean corpuscular hemoglobin concentration, MCV = mean corpuscular volume, NLR = neutrophil lymphocyte ratio, a = *t*-test or the Mann-Whitney *U* test for numeric variables, chi-square test or Fisher's exact test for categorical variables.

**Fig. 1:**
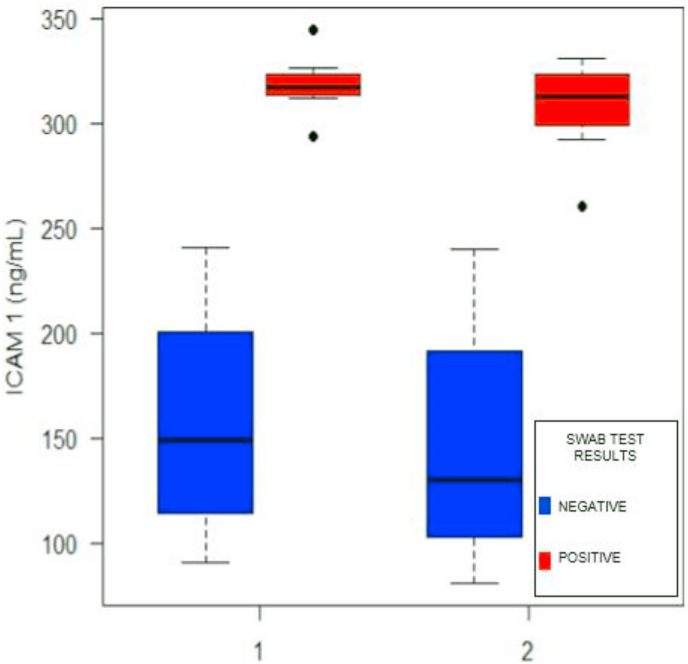
The relationship between serum ICAM-1 level and RT-PCR COVID-19 swab test results based on time of examination, pre- (1) and post- (2) ulcer debridement. P-value for repeated-measure ANOVA for swab test result group <0.001, time <0.001, and interaction = 0.721.

Table 2 compares the relationship between serum ICAM-1 levels in the COVID-19 and non-COVID-19 groups before and after debridement and with other COVID-19 criteria, such as high NLR value, pneumonia, leukopenia or leucocytosis, and lymphocytopenia. Regardless of the time of examination (before or after debridement), serum ICAM-1 levels differed between patients in the swab-positive and negative groups. There were notable differences in ICAM-1 levels between the COVID-19 and non-COVID-19 groups at baseline and even after debridement (p < 0.001). However, the differences in ICAM-1 levels before and after debridement in each group were non-significant. Remarkable differences were also seen in the NLR values (p = 0.001) and leukocyte counts (p = 0.042). The results in Fig. 1 mirror those presented in Table 2, where ICAM-1 levels were significantly elevated in the COVID-19 group relative to the non-COVID-19 group, before and after debridement. The other criteria in Table 2 did not correlate significantly, except for the association between pneumonia and the results of the RT-PCR COVID-19 swab test. However, some patients with negative swab tests were also diagnosed with pneumonia during the treatment.

**Table 2:** The relationship between serum ICAM-1 levels and screening criteria for COVID-19 and RT-PCR swab test results.

Variable	n (%)	Total (N = 20)	n (%)	Negative (n = 12)	n (%)	Positive (n = 8)	_p_a
		Med (Q_1_; Q_3_)		Med (Q_1_; Q_3_)		Med (Q_1_; Q_3_)	
Pre-debridement	*	222.6 (142.9; 315.0)	*	149.2 (116.8; 197.0)	*	317.2 (314.1; 321.9)	<0.001
Post-debridement	*	213.8 (120.8; 307.3)	*	130.3 (103.1; 185.1)	*	312.5 (303.1; 321.8)	<0.001
Difference	*	−7.3 (−18.6; −3.8)	*	−7.3 (−15.4; −4.7)	*	−5.4 (−20.8; 0.1)	0.427
Neutrophil Lymphocyte Ratio							
≤3,13	7 (35)	*	4 (33)	*	3 (38)	*	1.000
>3,13	13 (65)	*	8 (67)	*	5 (62)	*	
With Pneumonia	14 (70)	*	6 (50)	*	8 ()	*	0.042
Leukocyte count (10^3^ = mL)							
5-10′	6 (30)	*	3 (25)	*	3 (38)	*	0.642
<5 or >10	14 (70)	*	9 (75)	*	5 (62)	*	
Lymphocyte count (%)							
≥15	15 (75)	*	8 (67)	*	7 (88)	*	0.603
<15	5 (25)	*	3 (33)	*	2 (12)	*	

Med = median, Q1 = Quartile I, Q3 Quartile III. ^a^*t*-test or Mann-Whitney *U* test for numeric variables, chi-square test or Fisher's exact test for categorical variables.

## 4 Discussion

A novel finding from our study is that ICAM-1 levels were significantly elevated in patients with COVID-19 with DFUs compared with patients without COVID-19 (Table 2 & Fig. 1). This suggests that ICAM-1 could be used as an additional biomarker of COVID-19 since inflammation plays a crucial role in the pathogenesis of the disease. Induced by inflammation, ICAM-1 facilitates leukocyte-endothelial binding and the migration of leukocytes across the endothelial barrier [21]. As such, elevated levels of ICAM-1 mark a pre-existing inflammation process. Moreover, a retrospective study in China of 39 patients with COVID-19 and 32 controls showed that the former had significantly increased levels of ICAM-1 and other endothelial adhesion molecules, which may contribute to coagulopathy [22]. COVID-19-associated coagulopathy seems to happen due to excessive levels of von Willebrand factor, platelet activation, and hypercoagulability [23]. Therefore, an acute inflammatory process followed by a hypercoagulable state can contribute to the severity of COVID-19.

Diabetes is one of many comorbidity risk factors for COVID-19. The pathological mechanism underlying diabetes, including chronic inflammation and coagulopathy, encourages COVID-19 progression. Inflammatory biomarkers such as IL-6, C-reactive protein, serum ferritin, coagulation index, and D-dimer are increased in patients with diabetes compared with those without diabetes [24]. Other studies also found higher levels of serum ICAM-1 in patients with diabetes compared with controls [25]. In patients with diabetes, hyperglycemia causes both the innate and adaptive immune responses to become dysregulated; the resulting impairment contributes to the systemic tissue damage, respiratory and multiorgan failure, and inability to defend against invading pathogens characteristic of the disease. A harmful cytokine storm develops more readily when patients with diabetes are presented with an immune insult due to their preexistent, chronic low-grade hyper-inflammatory state. Moreover, particularly in cases with a high viral load, the capacity to mount an effective acute immune response against SARS-CoV-2 might be compromised in these patients, thereby rendering them more susceptible to the severe adverse effects associated with COVID-19 [24]. Moreover, hyperglycemia stimulates the production of coagulation factors such as von Willebrand factor, factor VII, factor VIII, factor IX, factor XII, the extrinsic pathway, and thrombin factor, which leads to excessive thrombus formation in the arteries, veins, and microvascular circulation [26].

A complication associated with diabetes is DFUs caused by peripheral neuropathy, reduced peripheral blood flow, and peripheral artery disease [[27], [28], [29], [30]]. DFU management requires a multidisciplinary approach including blood glucose control, proper wound treatment, and eliminating risk factors that may hinder or delay the wound healing process. However, patients with COVID-19 are subjected to a severe systemic inflammatory response and hypercoagulability that further compromises the blood supply to the ulcer and adds additional burden to the ongoing infection by prolonging the inflammation, hence worsening the DFU outcome [27].

Albumin is an essential protein in the wound healing process [31]. Low albumin levels are associated with inflammation and are often found in difficult-to-heal diabetes-associated wounds [32,33]. Our findings support this theory as low albumin levels were found in both patient groups (Table 1). In addition, an observational study of patients with COVID-19 in Italy found that severe hypoalbuminemia is positively correlated with COVID-19 mortality (HR: 2.48, 95% CI: 1.44–4.26; p = 0.001) and is also associated with old age (R = −0.367; p < 0.001) [34]. In their historical prospective study, Akirov et al. also correlated hypoalbuminemia with mortality among hospitalized patients diagnosed with several comorbidities including malignancy, ischemic heart disease, and diabetes mellitus [35]. Unfortunately, the correlation between COVID-19 severity and low albumin levels were not analyzed in this study due to its small sample size and limited data collection period, which are the limitations of this study. Therefore, further research is needed to identify other parameters associated with COVID-19 that enable early diagnosis and, ideally, accurate prognosis.

## 5 Conclusions

The statistically significant increase in ICAM-1 serum levels among patients with COVID-19 compared with their non-COVID-19 counterparts supports the inclusion of this metric as a preliminary screening method for SARS-CoV-2 infection particularly for asymptomatic patients prior to surgery or other medical procedures to enable healthcare workers to take the proper precautions when providing care for these patients.

## Declaration of competing interest

The authors declare that they have no conflict of interests.

## Registration of research studies

This study has been registered with the Research Registry no. 6341. https://www.researchregistry.com/register-now#home/registrationdetails/5fcce2e1748734001c96ba4c/
